# Medical Items

**Published:** 1894-03

**Authors:** 


### MEDICAL ITEMS.

"What is the subscription price of your journal?" "Two dollars a year." "Is it intended for any particular class of doctors?" "Yes; it's for those who have two dollars."

At a late examination in this city, Professor W. asked:

"Mr. D., how do hairpins and other foreign bodies get into the bladder?"

Mr. D.—"Suppose they are swallowed."

Germany, with a population of 50,000,000, had 21,620 physicians in 1893, or one physician to 2,314 people. The United States, with a population of 65,000,000, has 105,000 physicians, or one physician to every 620 people. In Georgia, with a population of 1,840,000, there are 2,700 doctors, or 1 to 680 people.

We have received the first number of Mathews' *Medical Quarterly*, which certainly makes a very promising beginning. It will be devoted mainly to the diseases and surgery of the rectum and intestines, but will have a value and interest to the general physician as well as the specialist. The first issue contains 188 pages of good matter, and is printed in an attractive style. The editor is Dr. Jos. M. Mathews, Louisville, Ky. Price, $2.00 per year.

Messrs. William Wood & Co., of New York, have in press a large treatise on Medical Jurisprudence and Toxicology. The work will be edited by Dr. R. A. Witthaus, Professor of Chemistry in the University of the City of New York, and Tracy C. Becker, Esq., Professor of Criminal Law and Medical Jurisprudence in the University of Buffalo. The editors have had the active assistance of several gentlemen high in authority in the medical and legal professions, and their work will doubtless be an able and thorough treatise. It will comprise four octavo volumes, and will be sold only by subscription. Price, five dollars per volume, in cloth.

FRANCE and Germany have lately lost two of their most prominent medical men. Paul Diday, M. D., known all over the world from his contributions to the literature of venereal diseases, died in Lyons, France, January 8th, in his eighty-third year. He had led an active life in his profession and was one of the founders of the *Lyons Medical,* one of the most valuable of European medical periodicals. The distinguished German surgeon, Theodor Billroth, died in Austria, February 6th. Professor Billroth had been for many years Professor of Surgery in the University of Vienna, and was the first (in 1881) to do a successful hysterectomy for cancer of the stomach. He had contributed a great deal to the literature of surgery, his work on "Surgical Pathology" being probably his best known.

